# Coexistence of t(15;17) and t(15;16;17) detected by fluorescence *in situ* hybridization in a patient with acute promyelocytic leukemia: A case report and literature review

**DOI:** 10.3892/ol.2014.2304

**Published:** 2014-07-02

**Authors:** RUI ZHANG, YOUNG-MI KIM, XIANFU WANG, YAN LI, HUI PANG, JI-YUN LEE, SHIBO LI

**Affiliations:** 1 Department of Pediatrics, University of Oklahoma Health Sciences Center, Oklahoma City, OK 73104, USA; 2 Department of Hematology, The First Affiliated Hospital of China Medical University, Shenyang, Liaoning 110001, P.R. China; 3 Department of Pathology, College of Medicine, Korea University, Seoul 136-705, Republic of Korea

**Keywords:** promyelocytic leukemia/retinoic acid α-receptor, array comparative genomic hybridization, variant translocation, acute promyelocytic leukemia, fluorescence *in situ* hybridization

## Abstract

Acute promyelocytic leukemia (APL) is characterized by the t(15;17)(q22;q21), which results in the fusion of the promyelocytic leukemia (*PML*) gene at 15q22 with the retinoic acid α-receptor (*RARA*) gene at 17q21. The current study presents the case of a 54-year-old female with APL carrying the atypical *PML/RARA* fusion signal due to a novel complex variant translocation t(15;16;17)(q22;q24;q21), as well as the classical *PML/RARA* fusion signal. Subsequent array comparative genomic hybridization revealed somatic, cryptic deletions on 3p25.3, 8q23.1 and 12p13.2-p13.1, and a duplication on 8q11.2; however, no genetic material loss or gain was observed in the breakpoint regions of chromosomes 15, 16 or 17. To the best of our knowledge, this is the first report of the coexistence of two abnormal clones, one classical and one variant, presenting simultaneously in addition to cryptic chromosome segmental imbalances in an adult APL patient.

## Introduction

Acute promyelocytic leukemia (APL) is characterized by particular clinical features that are important in differentiating it from other acute myeloid leukemias (AMLs) and determining an accurate diagnosis. These clinical features include unique hemorrhagic syndrome, disseminated intravascular coagulation (DIC) and association with the translocation between chromosomes 15 and 17, resulting in the formation of two reciprocal fusion genes; promyelocytic leukemia (*PML*)/retinoic acid α-receptor (*RARA*) on chromosome 15 and *RARA/PML* on chromosome 17. These fusion genes are sensitive to retinoid differentiating agents, such as all-trans retinoic acid (ATRA), and novel antiapoptotic agents, including arsenic trioxide (1,2).

Although t(15;17) has been found in ~90% of APL patients, variant translocations have been reported in a few APL patients, which are described as simple translocations involving chromosome 15 or 17 with any other chromosomes [t(15;v) or t(17;v)] or complex translocations characterized by the involvement of additional chromosome(s) in addition to chromosomes 15 and 17 [ct(15;17;v)] (3–5). In the last few years, a number of studies have focused on the alternate translocation in APL, for example t(5;17)(q35;q21) forming *NPM/RARA*, t(11;17)(q23;q21) producing *PLF/RARA* fusion and t(11;17)(q13;q21) generating *NUMA/RARA*, providing advanced insights into the pathogenesis of APL (3,6,7). However, little is known concerning the complex variant translocations in APL. The current study reports a patient who presented with the classical t(15;17) and complex variant t(15;16;17)(q22;q24;q21), which were demonstrated by traditional cytogenetic analysis, including G-banding karyotype and fluorescence *in situ* hybridization (FISH). In addition, cryptic losses on 3p25.3, 8q23.1 and 12p13.2-p13.1, and a gain of chromosome 8q11.2, were revealed in the level of array comparative genomic hybridization (CGH). This study was approved by the Institutional Review Board (IRB) at the University of Oklahoma Health Sciences Center (IRB no. 13100; Oklahoma City, OK, USA). The patient provided consent.

## Case report

### Clinical presentation

The current study presents a 54-year-old female with APL who was admitted to the University of Oklahoma Health Sciences Center due to fever and bleeding gums. The peripheral blood examination showed a hemoglobin count of 7.1 g/dl (normal range, 12–15.5 g/dl) and platelet count of 35×10^3^/μl (normal range, 150–450×10^3^/μl), as well as a white blood cell count of 11.95×10^3^/μl (normal range, 3.5–10.5×10^3^/μl) with 86% blasts, characterized by small to large cells with irregular, lobulated and bilobed nuclei with prominent nucleoli. The blast cells in the peripheral blood exhibited scant to moderate rare blue granulated cytoplasm and the bone marrow aspirate revealed no cellular particles. The touch preparations showed suboptimal cellular morphology, but revealed numerous blasts that were more frequently granulated than those present in the peripheral smear. The core biopsy of the bone marrow revealed a cellularity of >95% consisting of sheets of immature cells. A diagnosis of APL, M3 variant was determined according to the French-American-British Cooperative Group criteria (8).

### Cytogenetics, FISH and array CGH analyses

**Figure 1:**
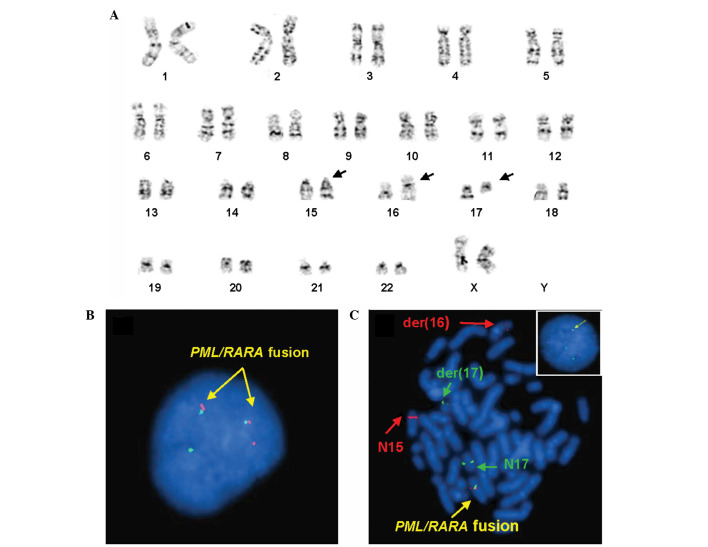
(A) Karyotype analysis revealed 46,XX,t(15;16;17)(q24;q24;q21) and the arrows indicate the t(15;16;17)(q24;q24;q21). Fluorescence *in situ* hybridization analyses using LSI PML (15q22; Spectrum Orange)/RARA (17q21; SpectrumGreen) dual color/dual fusion probe revealed a (B) typical PML/RARA dual color dual fusion signal and (C) variant t(15;17)(q22;q21) in the metaphase and interphase cells. PML/RARA, promyelocytic leukemia/retinoic acid α-receptor; N, normal; der, derivative.

**Figure 2:**
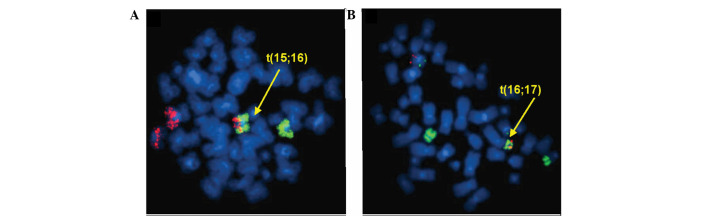
Fluorescence *in situ* hybridization analyses using cohybridization of (A) WCP15 (SpectrumOrange) with WCP16 (SpectrumGreen) showed t(15;16) and (B) WCP17 (SpectrumGreen) with subtelomeric probe for chromosome 16p (SpectrumGreen) and q (SpectrumOrange) showed t(16;17). WCP, whole chromosome painting probes.

The karyotype analysis at diagnosis revealed that the 20 metaphases analyzed exhibited a variant translocation among three chromosome; chromosomes 15, 16 and 17. The karyotype was designated as 46,XX,t(15;16;17)(q24;q24;q21)[20] (Fig. 1A). FISH analyses using the LSI PML/RARA dual color and dual fusion probe (Abbott Molecular, Inc., Des Plaines, IL, USA) was applied to the uncultured and cultured cells. On uncultured interphase cells, 12 out of 200 cells (6%) exhibited two classical *PML/RARA* fusion signals, one SpectrumOrange and one SpectrumGreen signal, which were the result of the classical t(15;17) (Fig. 1B) (http://www.abbottmolecular.com/us/siteMap.html#sthash.483IvlrV.dpuf). While 168 out of 200 cells (84%) exhibited one fusion signal, two SpectrumOrange signals and two SpectrumGreen signals, which were the result of the complex variant translocation, t(15;16;17) (Fig. 1C) (9). By combining the karyotyping and FISH results, it was demonstrated on metaphase cells that a part of the PML gene labeled with SpectrumOrange was present on the derivative chromosome 16 and a part of the RARA gene labeled with SpectrumGreen signal was present on the derivative chromosome 17 (Fig. 1C). Subsequent cohybridization of whole chromosome painting probes (WCP) 15 and 16 (Cytocell, Ltd., Cambridge, UK) and cohybridization of WCP17 (Abbott Molecular, Inc.), as well as the subtelomeric probes for chromosome 16p and q (Abbott Molecular, Inc.) on metaphase cells, confirmed the complex translocations among chromosomes 15, 16 and 17 (Fig. 2A and B) (http://www.abbottmolecular.com/us/siteMap.html#sthash.483IvlrV.dpuf) (10).

**Figure 3:**
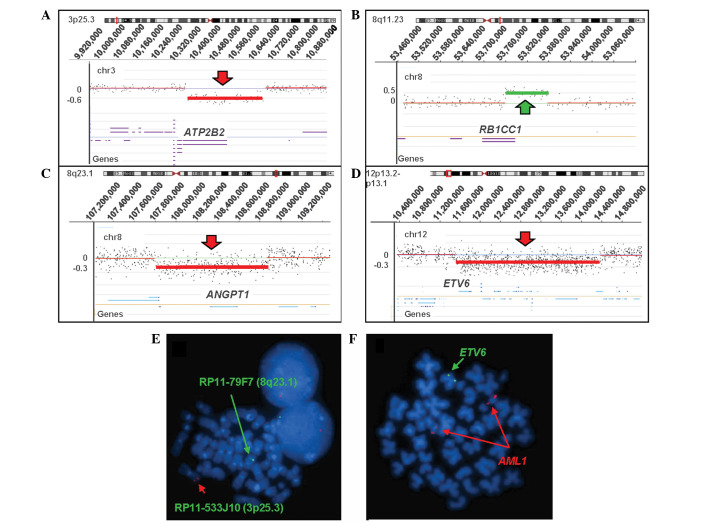
Results of the oligoarray CGH using NimbleGen SegMNT and the RefSeq genes in the abnormal region (University of California, Santa Cruz genome browser hg18). The Y-axis indicates a gain or loss of genetic material, while the X-axis indicates the genomic position of each feature on the chromosome. The red arrow indicates loss and the green arrow indicates gain and the significant gene in the lost or gained region is listed. (A) Loss of 3p25.3 (10,364,050–10,670,236 bp, hg18; ~0.3 Mb). (B) Gain of 8q11.23 (53,762,025–53,876,752bp, hg18; ~0.1 Mb), which is potentially constitutional rather than a disease-related duplication. (C) Loss of 8q23.1 (107,811,331–108,868,110 bp, hg 18; ~1.1 Mb). (D) Loss of 12p13.2-p13.1 (11,619,439–14,472,130 bp, hg18; ~2.9 Mb). Fluorescence *in situ* hybridization analyses using bacterial artificial chromosome RP11-533J10 (3p25.3; SpectrumOrange), RP11-79F7 (8p23.1; SpectrumGreen) and LSI ETV6 (SpectrumGreen) confirmed the array CGH results of the losses of (E) 3p25.3 and 8q23.1, and (F) ETV6 gene. CGH, comparative genomic hybridization; ETV6, ETS variant 6; AML1, acute myeloid leukemia-1.

Array CGH analysis showed the acquired genomic aberrations. No loss or gain was identified on chromosomes 15, 16, or 17, but cryptic losses of genomic material on 3p25.3, 8q23.1 and 12p13.1-p13.2, and a cryptic gain of 8q11.23 were detected (Fig. 3A–D). Several cancer-related genes were located in these genomic imbalanced regions. A loss of 3p25.3 (10,364,050–10,670,236 bp hg18; ~0.3 Mb) encompasses the plasma membrane Ca^2+^-ATPase 2 (*ATP2B2*) gene, a loss of 8q23.1 (107,811,331–108,868,110 bp hg18; ~1.1 Mb) contains the angiopoietin 1 (*ANGPT1*) gene, a loss of 12p13.2-p13.1 (11,619,439–14,472,130 bp hg18; ~2.9 Mb) involves ~23 genes, including the ETS variant 6 (*ETV6*) gene which is highly associated with t(12;21) in acute lymphoblastic leukemia, and a gain of 8q11.23 (53,762,025–53,876,752 bp hg18; ~0.1 Mb) contains the tumor suppressor gene retinoblastoma 1 inducible coiled-coil 1 (*RB1CC1*). Subsequent FISH confirmed the array CGH observations. The cultured cells were hybridized with home-brew probes using bacterial artificial chromosome clones, RP11-533J10 labeled with SpectrumOrange on 3p25.3 and RP11-79F7 labeled with SpectrumGreen on 8q23.1 (Invitrogen Life Technologies, Carlsbad, CA, USA), and 194 out of 200 cells (97%) presented with one SpectrumOrange signal and 104 out of 200 cells (52%) showed one SpectrumGreen signal, demonstrating the deletions of 3p25.3 and 8q23.1, respectively (Fig. 3E). The percentage of the cells with deletions was close to the percentage of cells with t(15;17) combined with t(15;16;17). These deletions are likely to be somatically acquired and not constitutional changes. In addition, hybridization with the LSI ETV6/AML1 dual color ES probe (Abbott Molecular, Inc.) confirmed the deletion of 12p13.1-p13.2 involving the *ETV6* genes (Fig. 3F) and 116 out of 200 (58%) cells were observed with deletion of the *ETV6* gene. However, gain of 8q11.23 was not be confirmed by FISH due to the small size (only 100 bp) of the duplication.

## Discussion

It is rare that complex variant translocations between chromosomes 15 and 17 [ct(15;17;v)] involving more than three chromosomes in APL result in the PML/RARA fusion gene. To the best of our knowledge, 45 cases have been reported (5,6,9,11–40), which account for 10% of APLs lacking classical t(15;17) (6). The clinical characterizations of the reported cases of APL with ct(15;17;v), including the present study, are summarized in Table I. No distinct clinical features have been observed in APL with ct(15;17;v) compared with APL with classical t(15;17). Generally, the ratio between males and females is 1:1, the median age at diagnosis is 45 years and 77% of ct(15;17;v) cases are accompanied with DIC. This is similar to the DIC ratio of cases with classical t(15;17). All observed ct(15;17;v) cases exhibited good responses to ATRA. Among these ct(15;17;v), nine recurrent breakpoints on the third or fourth chromosome consisted of the ct(15;17;v), including 1p36, 2q21, 3p21, 4q21, 11q13, 18q12, 20p13, 22q11.2 and Xq13. The current study presents the third case of APL with ct(15;17;v) involving chromosome 16. The first was described in a human immunodeficiency virus patient with secondary APL and the second was described in an APL patient with four chromosome translocations involving chromosomes 5, 15, 16 and 17 (23,29). However, the breakpoint of chromosome 16 in the current case (16q24) was different from those identified in the two previously reported cases. The breakpoint 16q24 has been described in AML with t(16;21)(q24;q22), which leads to the fusion gene *RUNX1/MTG16* and is predominantly associated with therapy-related AML (41). Whether the *MTG16* gene is located at the breakpoint and forms a fusion gene in the present case requires clarification.

In addition, the association between classical t(15;17) and ct(15;17;v) clones remains unclear. Several previous studies have assumed that the complicated rearrangements originate from the single standard translocation and then quickly outgrow this stem line (16,26,33). The current study is the first to report the presentation of two abnormal clones, classical t(15;17) and ct(15;17;v), simultaneously in an adult APL patient, providing indirect evidence that the variant translocation possibly evolves from the classical t(15;17).

It is well known that chromosomal imbalances, including the deletion(s) or amplification(s) of key driver gene(s), may promote the malignant transformation of leukemia (42–44). In the reported 45 APL cases of ct(15;17;v), eight cases presented with trisomy 8 or partial trisomy, including 8p+, 4p+, 9p+, +10 and +X. However, deletions, particularly small or cryptic deletions, have not yet been reported. In the present study, array CGH revealed the cryptic chromosome aberrations, including the deletions of *ATP2B2, ANGPT1* and *ETV6,* and a gain of *RB1CC1* genes, but no imbalances of the breakpoints of chromosomes 15, 16 or 17. FISH analysis not only confirmed the deletions of *ATP2B2, ANGPT1* and *ETV6*, but also demonstrated that these deletions were somatically acquired events since normal cells coexist with abnormal cells, which may potentially be involved in the leukemogenesis. However, the gain of 8q11.23 has not been confirmed due to the one size of duplication, which is too small to confirm by FISH. Furthermore, the possibility of a constitutional gain rather than disease-related gain cannot be ruled out. A divergence of ratio has also been identified between the deletions of *ATP2B2* (97%), *ANGPT1* (52%), *ETV6* (90%) and the PML/RARA fusion (classical and variant combined; 90%) by FISH, suggesting that these unbalanced genetic events may occur asynchronously in leukemogenesis.

In conclusion, the current study was the first to identify the classical t(15;17) and complex variant t(15;16;17) in an adult patient with APL using FISH. Furthermore, cryptic genomic alterations involving leukemia-related genes, such as *ATP2B2, ANGPT1, ETV6* and *RB1CC1*, were inferred in the level of array CGH and confirmed by FISH. It may be proposed that the malignant transformation of APL with complex variant translocation presents the following multi-step progression: i) Classical translocation; ii) formation of complex variant translocation by third chromosome involvement; and iii) further genomic changes. The further genomic changes, with the exception of the fusion of PML/RARA in APL, may be implicated in the heterogenicity of therapy outcome. Advanced study on these genes is likely to aid the elucidation of the oncogene or tumor suppressor gene candidates that potentially affect the prognosis of APL.

## Figures and Tables

**Table I:** Clinical characterization of reported acute promyelocytic leukemia cases with variant t(15;17).

Author (ref.)	Cytogenetic abnormality	Gender	Age, years	FAB	DIC	Cell clones	Treatment	Relapse	Survival, months
Berger *et al* (11)	t(15;17;4)(q22;q12;q21)	M	NA	M3	NA	46,XY, t(15;17;4)(q22;q12;q21)	CNA	NA	
Bernstein *et al* (12)	t(2;15;17)(q21?;q25?6?;q21?2?)	F	23	M3v	±	46,XX 46,XX,t(2;15;17)( q21?;q25?6?;q21?2?) 9p+	C +	6.5	
Bernstein *et al* (13)	t(3;15;17)(p21;q25?6?;q21?2?)	M	28	M3	+	46,XY 46,XY,t(3;15;17)( p21;q25?6?;q21?2?) 4p+	CNR	0.2	
Ohyashiki *et al* (14)	t(1;5;15;17)(p36;q31;q22;q12)	F	34	M3	+	46,XX 47,XX,+X, t(1;5;15;17)(p36;q31;q22;q12)	CNR	1	
Callen *et al* (15)	t(X;15;17)(q13;q21;q21)	F	48	M3v?	−	46,XX/46,XX, t(X;15;17)(q13;q21;q21) Relapse: 46,XX, t(X;15;17)(q13;q21;q21)	C +	13	
Bjerrum *et al* (16)	t(2;17;15)(q21;q21;q22or23or24)	M	10	M3	±	46,XY, t(2;17;15)(q21;q21;q22or23or24) 46,XY,t(15;17)(q22or23or24;q21)	C +	34	
						46,XY, t(2;17;15)(q21;q21;q22or23or24),8p+	A −	10+	
Osella *et al* (17)	t(1;15;17)(p36;q22;q21.1)	M	46	M3	+	46,XY 46,XY, t(1;15;17)(p36;q22;q21.1) 46,XY, t(1;15;17)(p36;q22;q21.1),+8			
Saitoh *et al* (18)	t(15;19;17)(q22;p13;q12)	F	16	M3	NA	46,XX 46,XX, t(15;19;17)(q22;p13;q12)	A −	7+	
Wan *et al* (19)	t(X;17;15)(q13;q12;q21)	F	30	M3	+	46,XX, t(X;17;15)(q13;q12;q21)	C −	2+	
Fujishima *et al* (20)	t(2;15;17)(q21;q22;q21)	M	77	M3	+	46,XY, t(2;15;17)(q21;q22;q21)	C+A	−	12+
Liu *et al* (21)	t(4;15;17)(q21;q22;q21)	M	37	M3	+	46,XY 46,XY, t(4;15;17)(q21;q22;q21)	As_2_O_3_	−	10+
Misawa *et al* (22)	t(7;17;15)(p22;q21.1orq12;q22)	M	35	M3	+	46,XY, t(7;17;15)(p22;q21.1orq12;q22)	C −	10+	
Zaccaria *et al* (23)	t(13;15;17;20)(q22;q22;q12;p13)	F	74	M3	+	46,XX 47,XX,+8, t(1;5;15;17)(p36;q31;q22;q12)	ANR	0.2	
	t(5;15;16;17)(q22;q22;p13;q12)	M	45	M3	+	46,XY 47,XY, t(5;15;16;17)(q22;q22;p13;q12)	C+A	+	24
Ogawa *et al* (24)	t(15;17;18)(q21q22;q12;q12)	M	53	M3	+	46,XY,del(15)(q21q22),der(17)t(17;18)(q12;q12), der(18)t(17;18)(q12;q12)ins(18;15)(q12;q21q22)	C −	3+	
Chen *et al* (25)	t(11;15;17)(q13;q22;q12)	F	15	M3	NA	46,XX, t(11;15;17)(q13;q22;q12)	NA	NA	NA
	t(11;15;17)(q13;q22;q12)	M	41	M3	NA	46,XY 46,XY, t(11;15;17)(q13;q22;q12)	NA	NA	NA
	t(5;15;17)(q13;q22;q12)	M	50	M3	NA	46,XY 46,XY, t(5;15;17)(q13;q22;q12)	NA	NA	NA
Calabrese *et al* (26)	t(15;17;21)(q22;q12;q22)	M	38	M3	NA	46,XY, t(15;17;21)(q22;q12;q22)	C+BMT	+	3
	der(4)t(4;17)(q11;p11),der(15)t(15;17)(q22;q21),der(17)t(15;17)(q22;q21)t(4;17)(q11;p11)	F	22	M3	NA	46,XX,der(4)t(4;17)(q11;p11),der(15)t(15;17)(q22;q21),der(17)t(15;17)(q22;q21)t(4;17)(q11;p11)	C+A	−	44+
Park *et al* (27)	t(1;15;17)(p31;q22;q21)	M	46	M3	+	46,XY			
						46,XY,del(1)(p22),del(3)(p25),der(17)t(1;15;17)(17pter→17q21::15q21→15q22::1p36→1p31::1 5q21→15q22::17q21→17pter)	C+A	+	9
Yamamoto *et al* (28)	t(15;20;17)(q22;p13;q21)	M	39	M3	+	46,XY 46,XY, t(15;20;17)(q22;p13;q21)	C+A	−	12+
Kudva *et al* (29)	t(15;16;17)(q22;q13;q21)	M	27	M3v	−	46,XY 46,XY, t(15;16;17)(q22;q13;q21)	C+A	−	40+
Eclache *et al* (30)	t(6;15;17)(q25;q22;q21)	F	56	M3	NA	46,XX 46,XX, t(6;15;17)(q25;q22;q21)	C+A	−	5+
Xu *et al* (5)	t(15;17) relapsed with t(4;15;17)(q27-28;q22;q21)	F	49	M3 M5a	NA	46,XX,dup(8)(q?),t(4;15;17)(q27-28;q22;q21)	C+A	+	10
García-Casado *et al* (31)	t(15;17(q22;q21),t(17;20)(q21;q12)	F	31	M3	−	46,XX			
						46,XX,der(15) t(15;17(q22;q21),t(17;20)(q21;q12)	C+A	−	60+
Yoo *et al* (32)	t(10;17;15;22)(q22;q21;q22;q11.2)	F	47	M3	NA	46,XX,t(10;17;15;22)(q22;q21;q22;q11.2)	C+A	−	1+
Miyazaki *et al* (33)	t(15;17) involved 8q21 and 14p?	M	78	M3v	−	46,XY 46,XY,t(15;17)(q22;q21) 46,XY,der(15)t(15;17)(q22;q21),der(17)(17pter→1 7q21::15q22→15qter::8q21→8qter) 46,XY,der(15)t(15;17)(q22;q21),der(17)(17pter→17q21::15q22→15qter::8q21→8qter::14p?→14qter)	C+A	−	14+
Abe *et al* (34)	t(5;17;15)(q11;q21;q22)	F	22	M3v	+	46,XX 46,XX, t(5;17;15)(q11;q21;q22)	C+A	−	12+
Stavropoulou *et al* (35)	t(15;17) ,t(17;18)	F	46	M3	NA	46,XX,der(15)t(15;17)(q22;q21),der(17)t(15;17)(q 22;q21)t(17;18)(q21;q12),der(18)t(15;17)(q22;q21)t(17;18)(q21;q12)	C+A	−	2
Kato *et al* (36)	t(15;22;17)(q22;q11.2;q21)	M	44	M3	+	46,XY 46,XY,t(15;22;17)(q22;q11.2;q21)	C+A	−	1+
Grimwade *et al* (6)	t(1;17;15)(p32;q21;q22)	M	NA	M3	NA	46,XY 46,XY, t(1;17;15)(p32;q21;q22) 46,idem,add(21)(p13)	NA	NA	NA
Grimwade *et al* (6)	t(7;17;15)(q22;q21;q22)	F	NA	M3v	NA	46,XX, t(7;17;15)(q22;q21;q22)	NA	NA	NA
	t(6;17;15)(p21;q21;q22)	M	NA	M3v	NA	46,XY 46,XY, t(6;17;15)(p21;q21;q22)	NA	NA	NA
	t(8;17;15)(q22;q21;q22)	F	NA	M3 and M3r	NA	46,XX 46,XX, t(8;17;15)(q22;q21;q22)47,idem,+8	NA	NA	NA
	t(13;17;15)(p13;q21;q22)	F	NA	M3v	NA	46,XX 46,XX, t(13;17;15)(p13;q21;q22)	NA	NA	NA
	t(5;17;15)(q14;q21;q22)	F	NA	M3	NA	46,XX,t(5;17;15)(q14;q21;q22 48,idem,+8,+21	NA	NA	NA
Tirado *et al* (36)	t(15;17;17)(q22;q23;q21)	F	52	M3	−	46,XX, t(15;17;17)(q22;q23;q21)	NA	−	12+
Freeman *et al* (37)	t(3;17;15)(q27;q21;q22)	M	78	M3	−	46,XY 46,XY, t(3;17;15)(q27;q21;q22)	C+A	−	NA
	t(8;17;15)(q24.3;q12;q22)	F	49	M3	+	46,XX 46,XX, t(8;17;15)(q24.3;q12;q22)	C+A	−	NA
Huret *et al* (38)	t(4;15;17)(q21;q26;q22) relapsed	M	67	M3	+	46,XY, 46,XY, t(4;15;17)(q21;q26;q22) 46,XY, add(1)(p?),t(1;18)(p36;p10),add(10)(p?), t(15;17)(q26;q22)	C	+	25+
McKinney *et al* (39)	t(3;17;15)(p21;q21;q22)	F	51	M3	+	46,XX,t(3;17;15)(p21;q21;q22)	C	+	33
	t(3;17;15)(p12;q21;q22)	M	63	M3	+	46,XY 46,XY, t(3;17;15)(q12;q21;q22)	C	+	14
Galieni *et al* (40)	t(1;17;15)(q23;q23;q22)	M	41	M3	NA	46,XY			
						46,XY, t(1;17;15)(q23;q23;q22)47,idem, +10	C+A	−	8+

FAB, French-American-British classification of acute leukemia; DIC: disseminated intravascular coagulation; NA, not available; NR, not remission; C, chemotherapy; A, all-trans retinoic acid; As_2_O_3_, arsenic trioxide; BMT, bone marrow transplantation; M, male; F, female.
